# Supplementary material for: The silicon cycle impacted by past ice sheets
Source: Nat Commun. 2018 Aug 10;9:3210. doi: 10.1038/s41467-018-05689-1 (PMC6086862; doi:10.1038/s41467-018-05689-1)
Supplement: Supplementary file 1 — Supplementary Information [file 41467_2018_5689_MOESM1_ESM.docx]

**Supplementary Information for “The global silicon cycle impacted by past ice sheets”**

Jon R Hawkings^1^*, Jade E Hatton^1^, Katharine R Hendry^2^, Jemma L Wadham^1^, Gregory F de Souza^3^, Ruza Ivanovic^4^, Tyler Kohler^5^, Marek Stibal^5^, Alexander Beaton^6^, Guillaume Lamarche-Gagnon^1^, Andrew Tedstone^1^, Mathis P Hain^7,8^, Elizabeth Bagshaw^9^, Jennifer Pike^9^, Martyn Tranter^1^

1. Bristol Glaciology Centre, School of Geographical Sciences, University Road, BS8 1SS, UK
2. School of Earth Sciences, University of Bristol, Bristol, BS8 1RJ
3. ETH Zurich, Department of Earth Sciences, Clausiusstrasse 25, 8092 Zurich, Switzerland
4. School of Earth and Environment, University of Leeds, Leeds, LS2 9JT, UK
5. Department of Ecology, Charles University in Prague, Viničná 7, 12844 Prague 2, Prague, Czech Republic
6. National Oceanography Centre, University of Southampton Waterfront Campus, European Way, Southampton, SO14 3ZH, UK
7. Earth and Planetary Sciences, University of California, Santa Cruz, CA 95064, USA
8. Ocean and Earth Science, National Oceanography Centre Southampton, University of Southampton, Waterfront Campus, European Way, Southampton, SO14 3ZH, UK
9. School of Earth and Ocean Sciences, Cardiff University, Main Building, Park Place, Cardiff, CF10 3AT, UK

* Correspondence to jon.hawkings@bristol.ac.uk

**Supplementary Figure 1. Leverett Glacier meltwater discharge over the 2015 melt season.** Proglacial meltwater river discharge was monitored from onset (28^th^ May) to the end of the ablation season (15^th^ September). Meltwater outburst events, in response to supraglacial forcing, are highlighted by red shading.

**
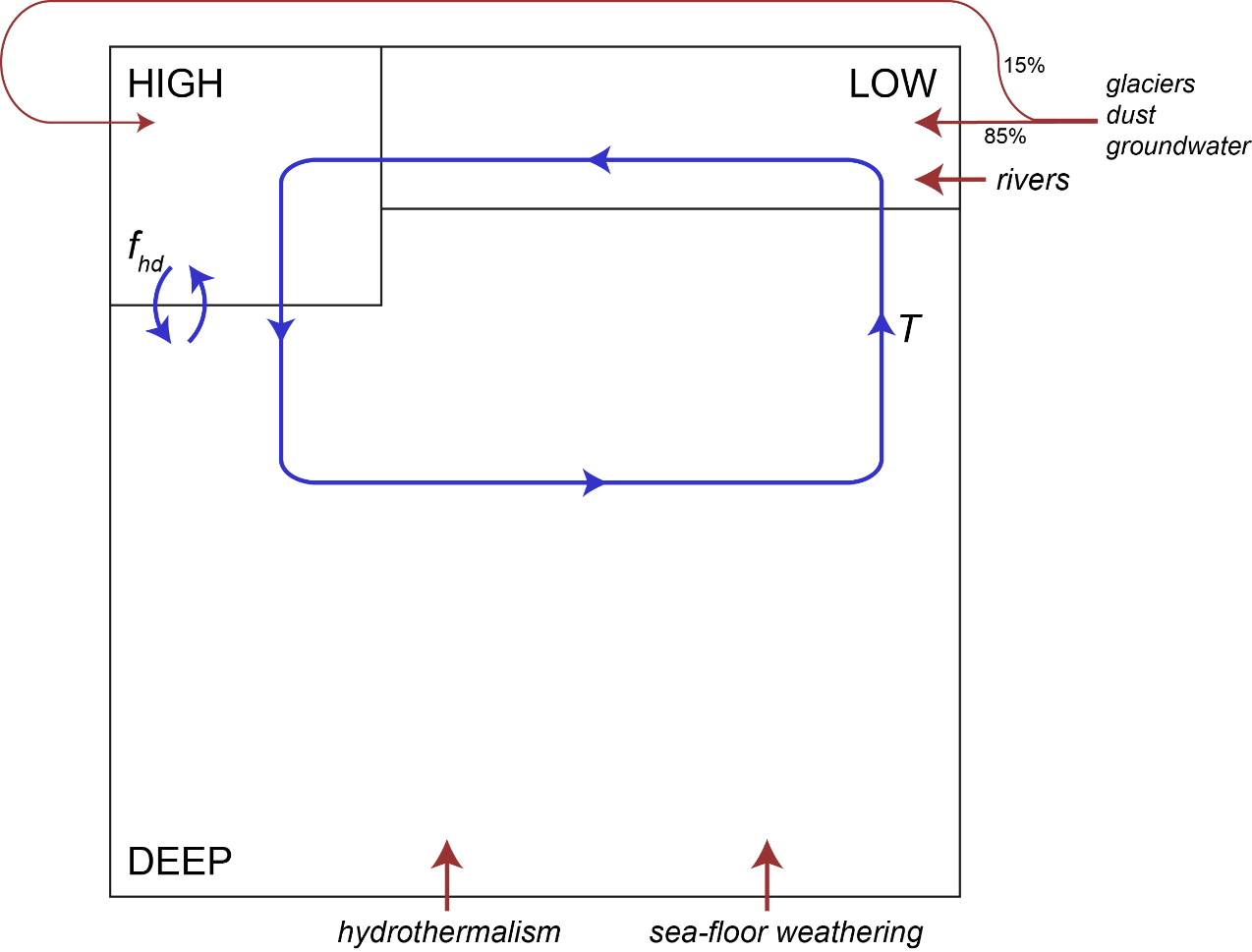
**

**Supplementary Figure 2. Schematic of three-box model.** Adapted from Sarmiento and Toggweiler ^1^. Blue arrows represent internal ocean circulation volume fluxes, brown arrows signify external inputs of Si to the ocean.

**Supplementary Figure 3. Long-term simulation of the 3-box model response to changed Si input fluxes.** Regardless of whether Si input fluxes lead to a change in whole-ocean Si concentration, the switch from glacial to riverine dominance produces a long-term change in whole-ocean δ^30^Si that exactly mirrors the change in the δ^30^Si of the inputs.


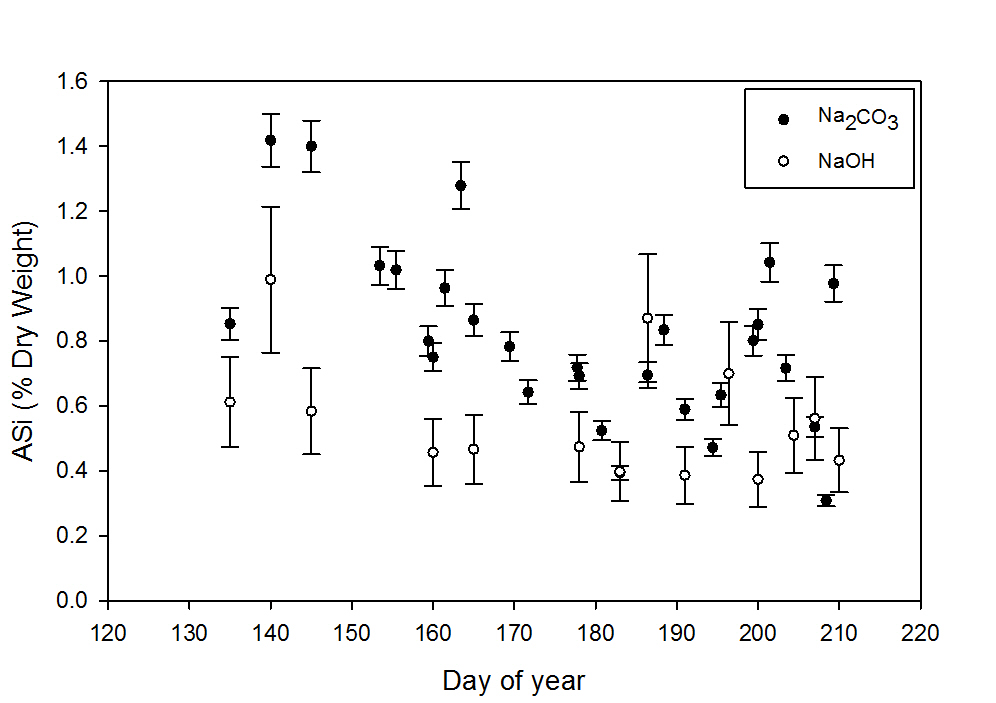


**Supplementary Figure 4. Comparison of measured ASi concentration with 0.1 M Na_2_CO_3_ and 0.2 M NaOH extraction methods**. Filled circles indicate concentrations measured after Na_2_CO_3_ extraction, with error bars representing mean replicate deviation of ±5.7%. Open circles indicate the concentrations with the NaOH extraction, with error bars representing mean replicate deviation of ±22.7%. Corresponding s.d. for all data points are show. Note that due to limited sediment quantities, not all samples could be matched.


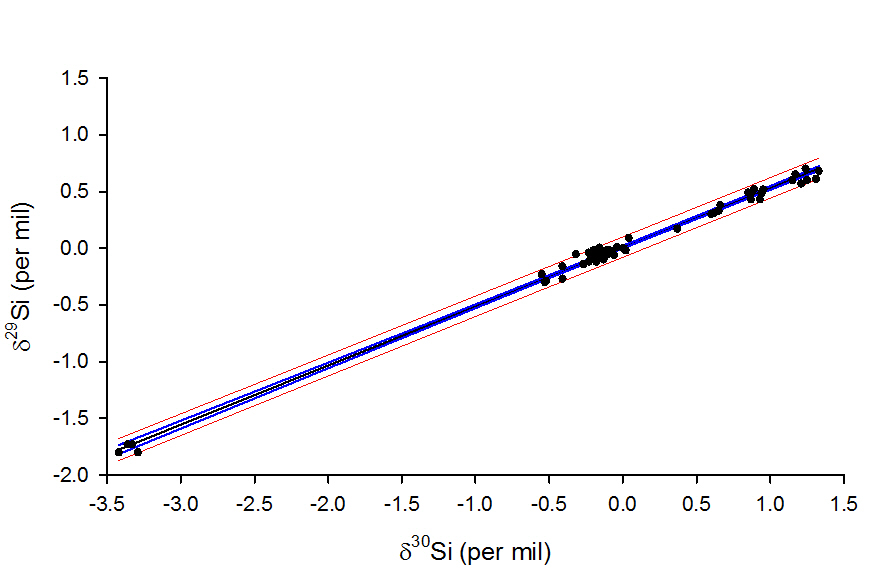


**Supplementary Figure 5. Three isotope plot of all samples from the study.** The black regression line has a gradient of 0.523 ±0.025 range, which shows mass-dependent fractionation. The blue lines represent 95% confidence intervals and the red lines represent 95% prediction intervals.


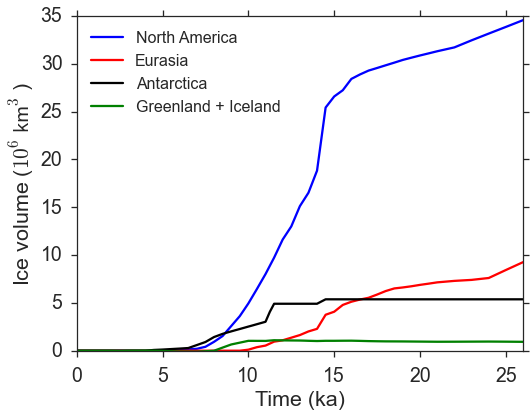


**Supplementary Figure 6.** **Change in the ice volume of the palaeo-ice sheets, Greenland ice sheet and the Antarctic ice sheet from the Last Glacial Maximum (LGM - ~26-21 ka) to present day (0 ka) ^2^.** Ice volume changes for different ice sheets are indicted by the coloured lines.

**
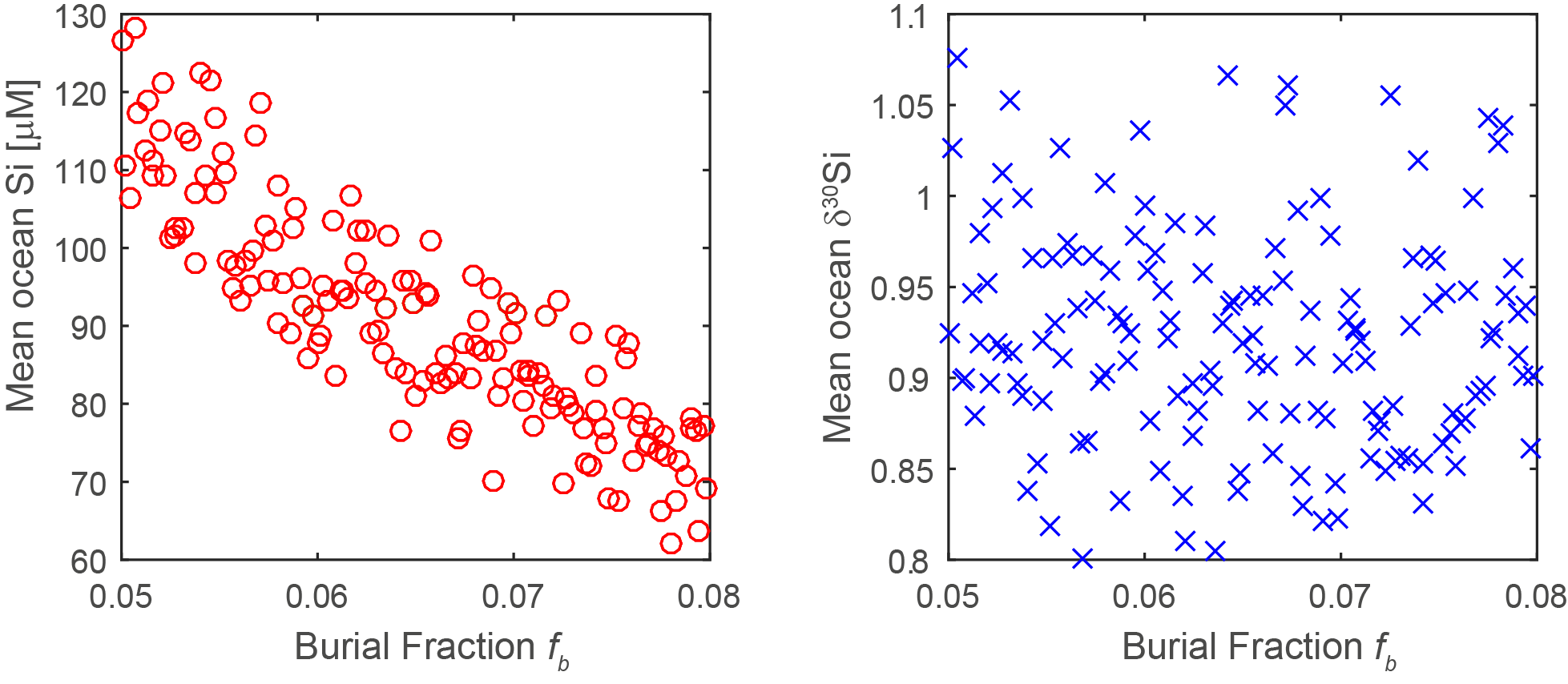
**

**Supplementary Figure 7. Systematics of a 150-member sensitivity ensemble**. Si input fluxes to the model were varied according to the range of uncertainty of the modern input flux (Frings et al., 2016). Burial fraction *f_b_* was varied concurrently between 0.05 and 0.08. As can be seen in the left panel, mean-ocean Si concentrations similar to the observed value of 92 μM are obtained for burial fractions centered around 0.065, with a range of ~0.055 to ~0.075. The right panel shows that mean-ocean δ^30^Si is insensitive to the numerical value of *f_b_*.

**Supplementary Table 1. Key parameters of the 3-box Si-cycling model**

| **Parameter** | **Value** | **Units** |
| --- | --- | --- |
| *Model architecture* |  |  |
| High-latitude exchange *f_hd_* | 38.1 | Sv |
| Overturning *T* | 25.4 | Sv |
| Total ocean volume | 1.35 × 10^18^ | m^3^ |
| *Si cycling parameters* |  |  |
| Whole-ocean [Si] | 92 | μM |
| Low-latitude uptake rate *k_l_* | 0.6 | yr^-1^ |
| High-latitude uptake rate *k_h_* | 0.225 | yr^-1^ |
| Burial fraction *f_b_* | 0.056 – 0.073 | - |
| Glacial DSi scaling *S_glac,DSi_* | 9.18 × 10^6^ – 5.69 × 10^7^ | mol Si / km^3^ |
| Glacial ASi scaling, *S_glac,ASi_* | 1.21 × 10^8^ – 1.06 × 10^9^ | mol Si / km^3^ |
| Riverine DSi scaling *S_riv,DSi_* | 1.52 × 10^8^ – 1.86 × 10^8^ | mol Si / km^3^ |
| Riverine ASi scaling *S_riv,ASi_* | 2.40 × 10^7^ – 7.83 × 10^7^ | mol Si / km^3^ |

**Supplementary Table 2. Summary of 3-box model inputs.** Magnitude of model inputs and associated δ^30^Si composition of inputs are shown at five select time points. Flux ranges expressed were used to constrain the Latin Hypercube sampling for each model run. Last Glacial Maximum (LGM) inputs were used to spin up the model to LGM conditions over a 100,000-year period. Values are expressed to three significant figures. See *Flux estimates for silicon isotope 3-box model* for further information on how these figures were derived and references.

|  | **Units** | **LGM**  **(ca. 21,000 ka)** | **MWP1a**  **(ca. 14,000-14,500 ka)** | **MWP1b**  **(ca. 11,000-11,500 ka)** | **Holocene**  **(ca. 8,000 ka)** | **Modern** |
| --- | --- | --- | --- | --- | --- | --- |
| ***Glacial input*** |  |  |  |  |  |  |
| **Runoff** | km^3^ year^-1^ | 7,740 | 21,600 | 12,000 | 5,830 | 1,400 |
| **DSi flux** | Tmol year^-1^ | 0.071 – 0.440 | 0.202 – 1.25 | 0.110 – 0.684 | 0.053 – 0.331 | 0.013 – 0.080 |
| **ASi flux** | Tmol year^-1^ | 0.935 – 8.18 | 2.66 – 23.2 | 1.45 – 12.7 | 0.704 – 6.16 | 0.169 – 1.48 |
| **δ^30^DSi** | ‰ | -0.37 – -0.13 | -0.37 – -0.13 | -0.37 – -0.13 | -0.37 – -0.13 | -0.37 – -0.13 |
| **δ^30^ASi** | ‰ | -0.27 – -0.15 | -0.27 – -0.15 | -0.27 – -0.15 | -0.27 – -0.15 | -0.27 – -0.15 |
|  |  |  |  |  |  |  |
| ***Non-glacial riverine input*** | | |  |  |  |  |
| **Non-glacial riverine input** | km^3^ year^-1^ | 28,100 | 29,900 | 33,900 | 36,700 | 37,300 |
| **DSi flux** | Tmol year^-1^ | 4.28 – 5.22 | 4.55 – 5.74 | 5.15 – 6.29 | 5.60 – 6.84 | 5.67 – 6.92 |
| **ASi flux** | Tmol year^-1^ | 0.675 – 2.20 | 0.718 – 2.35 | 0.814 – 2.65 | 0.884 – 2.88 | 0.895 – 2.92 |
| **δ^30^DSi** | ‰ | 0.57 – 1.93 | 0.57 – 1.93 | 0.57 – 1.93 | 0.57 – 1.93 | 0.57 – 1.93 |
| **δ^30^ASi** | ‰ | -0.43 – 0.07 | -0.43 – 0.07 | -0.43 – 0.07 | -0.43 – 0.07 | -0.43 – 0.07 |
|  |  |  |  |  |  |  |
| ***Other inputs*** |  |  |  |  |  |  |
| **Aeolian dust Si flux** | Tmol year^-1^ | 0.25 | 0.25 | 0.25 | 0.25 | 0.25 |
| **Aeolian dust δ^30^Si** | ‰ | -0.65 | -0.65 | -0.65 | -0.65 | -0.65 |
|  |  |  |  |  |  |  |
| **Groundwater**  **Si flux** | Tmol year^-1^ | 0.65 | 0.65 | 0.65 | 0.65 | 0.65 |
| **Groudwater δ^30^Si** | ‰ | 0.19 | 0.19 | 0.19 | 0.19 | 0.19 |
|  |  |  |  |  |  |  |
| **Hydrothermal Si flux** | Tmol year^-1^ | 0.6 | 0.6 | 0.6 | 0.6 | 0.6 |
| **Hydrothermal δ^30^Si** | ‰ | -0.3 | -0.3 | -0.3 | -0.3 | -0.3 |
|  |  |  |  |  |  |  |
| **Sea floor weathering flux** | Tmol year^-1^ | 0.4 | 0.4 | 0.4 | 0.4 | 0.4 |
| **Sea floor weathering δ^30^Si** | ‰ | -0.3 | -0.3 | -0.3 | -0.3 | -0.3 |

**Supplementary Table 3. Summary of Leverett Glacier silicon isotope results from 2015 melt season.** Data points selected to correspond with silicon isotope results.

| **Julian Day** | **Dissolved Silica δ^30^Si (‰)** | **Errors for Dissolved Silica (2σ S.D. ‰)** | **Total Reactive Silica δ^30^Si (‰)** | **Errors for Total Reactive Silica (2σ S.D. ‰)** | **Bulk Suspended Sediment**  **δ^30^Si (‰)** | **Q**  **(m^3^S^-1^)** | **DSi  (μmol L^-1^)** | **ASi**  **(%)** | **pH** |
| --- | --- | --- | --- | --- | --- | --- | --- | --- | --- |
| 122.4 | 1.31^a^ | 0.08 | - | - | - | - | - | - | - |
| 128.4 | 0.85 | 0.04 | - | - | - | - | 53.66 | - | 8.98 |
| 135.4 | 0.87 | 0.04 | 0.05 | 0.05 | - | - | 36.01 | 0.85 | 7.88 |
| 140.8 | - | 0.03 | 0.21 | 0.03 | - | - | 38.86 | 1.41 | 8.35 |
| 145.4 | 0.65 | 0.02 | -0.16 | 0.03 | - | - | 38.47 | 1.40 | 7.90 |
| 148.4 | - | - | - | - | 0.02 | 5.4 | 35.63 | - | 7.72 |
| 153.4 | 0.66 | 0.04 | - | - | - | 13.0 | 37.00 | 1.03 | 7.85 |
| 160.4 | *0.60 | 0.02 | - | - | -0.17 | 17.5 | 31.51 | 0.75 | 7.80 |
| 165.4 | 0.37 | 0.03 | -0.19 | 0.03 | -0.11 | 25.1 | 24.13 | 0.86 | 7.64 |
| 171.4 | 0.02 | 0.08 | - | - | - | 73.2 | 20.24 | 0.64 | 8.01 |
| 174.7 | -0.23 | 0.02 | - | - | - | 90.0 | 15.05 | - | 7.63 |
| 178.4 | -0.18 | 0.03 | -0.20 | 0.04 | - | 122.7 | 24.85 | 0.69 | 8.41 |
| 183.4 | -0.52 | 0.02 | -0.15 | 0.03 | -0.1 | 215.1 | 27.69 | 0.39 | 9.18 |
| 186.4 | -0.41 | 0.03 | - | - | - | 207.2 | 19.75 | 0.70 | 8.33 |
| 189.4 | -0.41 | 0.03 | - | - | - | 294.5 | 17.79 | 0.83^b^ | 8.76 |
| 191.4 | -0.22 | 0.03 | -0.27 | 0.04 | - | 360.9 | 21.41 | 0.59 | 9.24 |
| 196.3 | -0.55 | 0.04 | - | - | - | 230.1 | 16.29 | 0.64^b^ | 8.58 |
| 200.4 | -0.14 | 0.03 | -0.32 | 0.03 | - | 311.3 | 19.70 | 0.85 | 8.86 |
| 207.4 | - | - | -0.16 | 0.04 | - | 301.9 | 22.02 | 0.54 | 9.53 |
| 208.4 | -0.09 | 0.03 | - | - | - | 270.5 | 24.83 | 0.31 | 9.62 |
| 210.3 | -0.06 | 0.03 | -0.11 | 0.03 | - | 236.3 | 23.02 | 0.97^b^ | 9.51 |

**^a^** sample taken at glacier portal, approximately 1km upstream from usual sampling site.

**^b^** results are +/- 24 hours of the sample date, due to limited sediment available for analysis.

**Supplementary References**

1. Sarmiento, J. L., Toggweiler, J. R. A new model for the role of the oceans in determining atmospheric PCO2. *Nature* **308,** 621 (1984).

2. Ivanovic, R. F. *et al.* Transient climate simulations of the deglaciation 21–9 thousand years before present (version 1) – PMIP4 Core experiment design and boundary conditions. *Geosci. Model Dev.* **9**(7)**,** 2563-2587 (2016).
